# Agrin and Synaptic Laminin Are Required to Maintain Adult Neuromuscular Junctions

**DOI:** 10.1371/journal.pone.0046663

**Published:** 2012-10-03

**Authors:** Melanie A. Samuel, Gregorio Valdez, Juan C. Tapia, Jeff W. Lichtman, Joshua R. Sanes

**Affiliations:** Department of Molecular and Cellular Biology and Center for Brain Science, Harvard University, Cambridge, Massachusetts, United States of America; Columbia University, United States of America

## Abstract

As synapses form and mature the synaptic partners produce organizing molecules that regulate each other’s differentiation and ensure precise apposition of pre- and post-synaptic specializations. At the skeletal neuromuscular junction (NMJ), these molecules include agrin, a nerve-derived organizer of postsynaptic differentiation, and synaptic laminins, muscle-derived organizers of presynaptic differentiation. Both become concentrated in the synaptic cleft as the NMJ develops and are retained in adulthood. Here, we used mutant mice to ask whether these organizers are also required for synaptic maintenance. Deletion of agrin from a subset of adult motor neurons resulted in the loss of acetylcholine receptors and other components of the postsynaptic apparatus and synaptic cleft. Nerve terminals also atrophied and eventually withdrew from muscle fibers. On the other hand, mice lacking the presynaptic organizer laminin-α4 retained most of the synaptic cleft components but exhibited synaptic alterations reminiscent of those observed in aged animals. Although we detected no marked decrease in laminin or agrin levels at aged NMJs, we observed alterations in the distribution and organization of these synaptic cleft components suggesting that such changes could contribute to age-related synaptic disassembly. Together, these results demonstrate that pre- and post-synaptic organizers actively function to maintain the structure and function of adult NMJs.

## Introduction

At the mammalian skeletal neuromuscular junction (NMJ), pre- and post-synaptic structures are highly differentiated and precisely aligned. This synapse forms in embryos, matures during an early postnatal period, and is remarkably stable in young adult mice [1]–[3]. Later in life, however, maintenance of the NMJ is compromised, and several structural alterations occur. By two years of age, NMJs in most muscles show marked dystrophies, including nerve terminal sprouting, fragmentation, and denervation [4]–[9]. Related patterns of progressive NMJ disassembly have also been documented in some diseases of the motor unit, including myasthenia gravis and amyotrophic lateral sclerosis [9]–[13]. These alterations presumably result from changes in the level or distribution of molecules that maintain synaptic structure and function in young adults. To date, however, the identity of such factors has remained obscure.

In seeking such factors, we considered molecules called synaptic organizers, which are already known to regulate the formation and maturation of the NMJ [14]–[16]. Several synaptic organizers are secreted by motor axon terminals or muscle fibers and are stably maintained in the basal lamina that runs through the synaptic cleft between the nerve terminal and postsynaptic membranes [17]–[21]. Of these, the two best characterized are agrin and the synaptic laminins.

Agrin is a nerve-derived proteoglycan that is secreted by motor axons and promotes aggregation of acetylcholine receptors (AChRs) in the postsynaptic membrane. Muscle cells also express agrin, but only motor neurons produce an alternatively spliced isoform called “z-agrin” that activates a postsynaptic receptor complex consisting of LRP4 and the receptor tyrosine kinase MuSK [22]–[25]. During the initial phases of development, z-agrin activates MuSK/LRP4 to both stabilizes aneurally-formed clusters of AChRs and induce formation of new clusters in the muscle membrane [26]–[30]. In the absence of all agrin isoforms or only z-agrin, postsynaptic differentiation of the NMJ does not proceed, and animals die shortly after birth [18], [19]. Presynaptic abnormalities are also evident in agrin mutant mice, likely because disruption of postsynaptic development prevents retrograde signaling from muscle to nerve.

In contrast to nerve-derived agrin, laminins are synthesized by muscles and promote presynaptic differentiation. Laminins are present in all basal laminas (BLs) and are heterotrimers of α, β, and γ subunits drawn from a total of 5α, 3β, and 3γ isoforms [31]. There are specific laminin heterotrimers in the synaptic cleft, laminins 221 (α2β2γ1), 421 (α4β2γ1), and 521 (α5β2γ1); extrasynaptic BL contains primarily laminin 211 (α2β1γ1) [32]. In laminin-β2 mutant mice all synaptic laminin heterotrimers are depleted, the NMJ fails to mature and animals die 2–4 weeks after birth due to a lack of proper neuromuscular transmission [17], [33]. Mice lacking laminin-α4 (*lama4^−/−^*) are viable but lack precise apposition of presynaptic neurotransmitter release sites (active zones) to postsynaptic depressions called junctional folds [20]. Mice lacking both laminins-α4 and -α5 also show presynaptic abnormalities as well as delayed postsynaptic maturation [34].

Here, we asked whether agrin and synaptic laminins are required only for synaptic development and maturation, or whether their continuous presence is also required for synaptic maintenance in adult NMJs. To bypass the neonatal lethality of agrin mutants, we used a conditional allele (*agrn^flox^*, ref [35]) to selectively delete agrin from a marked subset of motoneurons in adults. Agrin deletion resulted in the depletion of AChR receptors, dispersion of basal lamina components, NMJ dystrophies, and eventually nerve retraction. Lacking a conditional laminin-β2 mutant, we used *lama4* mutants, which show subtle developmental effects but are otherwise normal and fertile [20]. By 6 months of age, NMJs lacking laminin-α4 exhibited severe structural defects. Abnormalities in both *agrn^flox^* and *lama4* mutants were distinct from each other, but resembled in several respects those seen in NMJs of aged wild-type mice. Moreover, the synaptic distribution of agrin and synaptic laminins changed with age. Together, these results suggest that maintenance of the NMJ requires the continuous presence of synaptic organizers and that alterations in the integrity of the synaptic basal lamina may contribute to age-related synaptic disassembly.

## Results

### Agrin Depletion by Conditional Deletion in Adults

**Figure 1:**
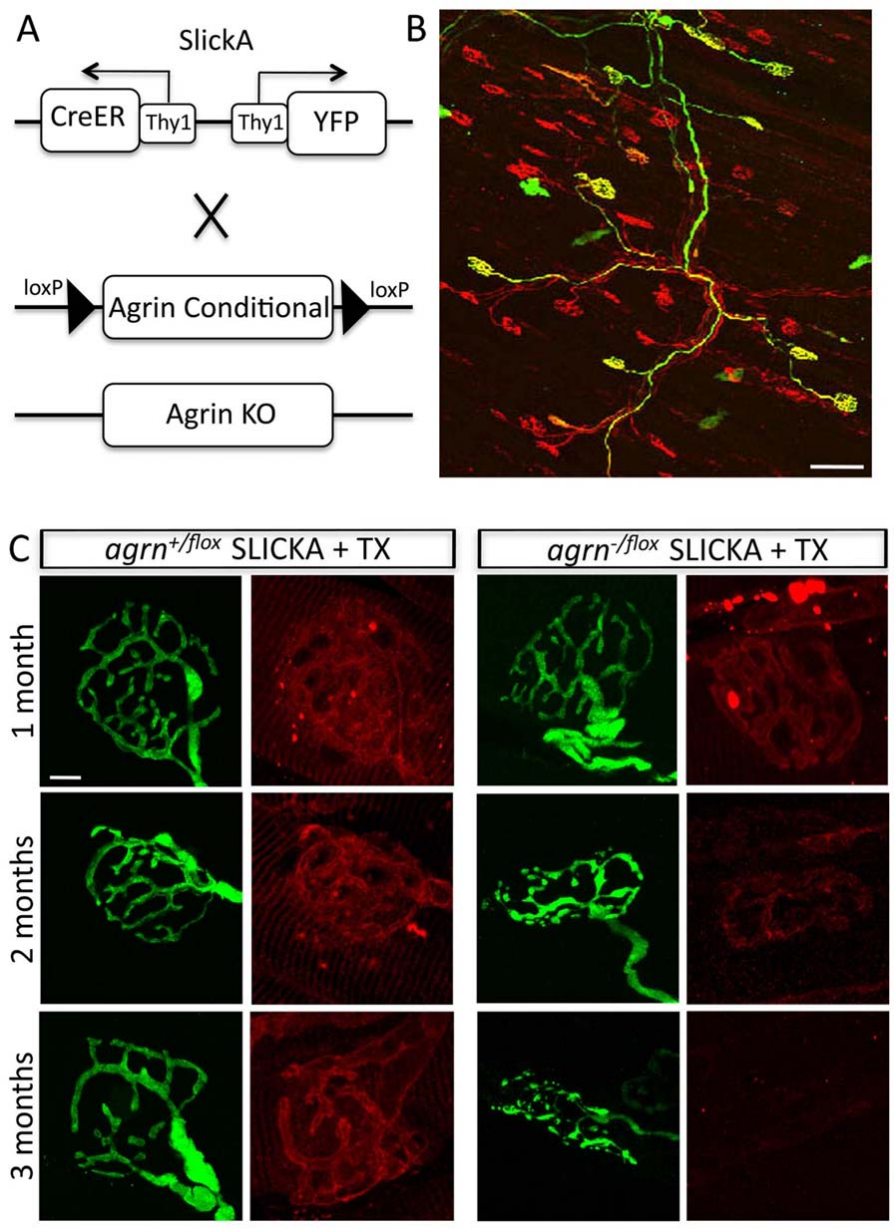
Agrin is depleted from NMJs following conditional deletion in adults. A. Schematic of the method used to delete agrin from subsets of motoneurons. In the SLICKA transgene, separate Thy1 regulatory elements drive expression of CreER and YFP. Following administration of tamoxifen, CreER is activated and the agrin gene is deleted in many YFP+ motoneurons. B. A YFP+ motor axon (green) in a triangularis sterni muscle. Unlabeled axons are stained with neurofilament and syntaptotagmin 2 (red) to mark all nerve terminals. Scale bar = 100 µm. Explain the red labeling of motor axons ***SLICK negative = Neurofilament/Syn??? C. Agrin at NMJs in control (*agrn^+/flox^*; SLICKA) and mutant (*agrn^−/flox^*; SLICKA) muscle at the indicated times after tamoxifen (TX) administration. Agrin levels are decreased slightly 1 month after TX and markedly by 3 months. Scale bar = 10 µm. TX = tamoxifen.

Mice lacking all agrin isoforms or only z-agrin die soon after birth due to severe neuromuscular defects [18], [19]. To bypass lethality, we used agrin conditional mutants (*agrn^flox^*) in which the genomic sequence from exon 6 to intron 33 is flanked by loxp sites [35]. For deletion in adults, we used a transgenic line termed SLICKA in which a tamoxifen-activated derivative of Cre recombinase (Cre-estrogen receptor fusion or Cre-er) and YFP are co-expressed in a subset of motor neurons under the control of regulatory elements from the *Thy1* gene [36]. Administration of tamoxifen deletes agrin from only those motoneurons that are YFP positive (YFP+) (**Fig. 1A, B**). In these experiments, we administered 8 doses of tamoxifen over 2 weeks [36] to maximize the fraction of YFP+ motoneurons in which agrin deletion occurred. Thus, many YFP positive motoneurons are agrin negative, and all YFP negative (YFP-) motor neurons are agrin positive, allowing comparison of wild-type and agrin-deficient NMJs in the same muscle. In the experiments presented here, we administered tamoxifen to 2–3 month old mice; to facilitate agrin deletion, experiments were performed with null/conditional trans-heterozygotes (*agrn^−/flox^*).

In initial studies, we assessed levels of agrin at the NMJ by immunohistochemistry 1 to 3 months post-tamoxifen injection. As expected, agrin was concentrated at NMJs in control muscle (**Fig. 1C**, left panel) and at YFP- NMJs in mutants (data not shown). In contrast agrin levels decreased at most YFP+ NMJs in mutants. Modest decreases could be observed as early as one month, but complete loss did not occur until 2 to 3 months after tamoxifen was administered (**Fig. 1C**, right panel). Nerve terminals were also altered; this result is discussed below.

### Agrin is Required for Maintenance of the Postsynaptic Apparatus

**Figure 2:**
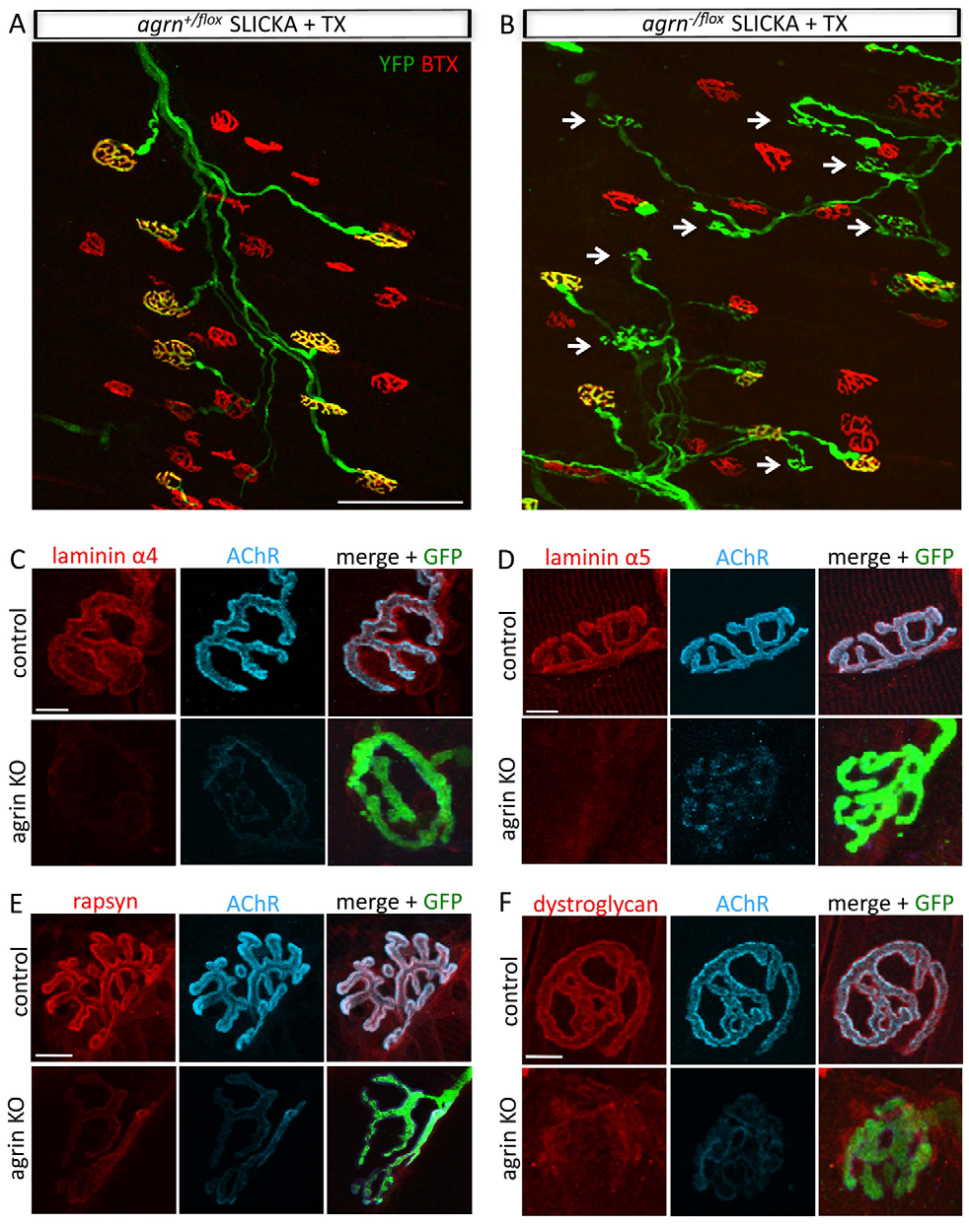
Agrin loss leads to disassembly of the postsynaptic apparatus. A–B. Triangularis sterni muscle from control (A) and conditional agrin knockout (B) animals 3 months after tamoxifen (TX) administration. TX administration to heterozygotes does not disrupt apposition of axons (green) to AChR-rich postsynaptic membrane (red), but many axons appose AChR-poor postsynaptic structure (arrows) in the mutant. Scale bar = 100 µm. TX = tamoxifen C–F Muscles from agrin conditional knockouts and controls 3 months after TX stained for laminin α4 (C), laminin α5 (D), rapsyn (E), and dystroglycan (F). In each case, mutant NMJs show markedly decreased levels of immunoreactivity. Scale bar = 10 µm.

The best-studied role of agrin is as a factor that promotes the stabilization of AChR clusters. To ask whether agrin is required for maintenance of these aggregates in adult muscle, we stained triangularis sterni muscle with fluorophore tagged α-bungarotoxin (f-BTX), which binds tightly and specifically to AChRs. In control heterozygous animals (*agrn^+/flox^*; SLICKA^+^) AChRs remained normally clustered 3 months after tamoxifen administration, and terminal branches of individual axons exactly opposed postsynaptic sites (**Fig. 2A**, left panel). In contrast, AChR receptor clusters were undetectable at a large number of YFP+ axon terminals in agrin mutants (**Fig. 2A**, right panel, arrows). Thus agrin is required for maintenance of AChR clusters at adult NMJs.

During development, agrin is required for most if not all aspects of postsynaptic maturation [18], [19]. To ask whether agrin is also required for the maintenance of the postsynaptic apparatus, we immunostained mutant muscles with antibodies to two components of the synaptic basal lamina (laminin-α4 and -α5), a component of the postsynaptic membrane (β-dystroglycan) and a component of the subsynaptic cytoskeleton (rapsyn). We focused on synapses that displayed structural defects as these show the highest level of agrin depletion (see below). Levels of laminin-α4 and -α5, both of which are concentrated in synaptic basal lamina at control NMJs, were markedly reduced in affected synapses (**Fig. 2C, D**). Likewise, levels of β-dystroglycan, a laminin receptor, and of rapsyn, a cytoskeletal protein that binds AChRs, were depleted (**Fig. 2E, F**). We also stained muscles with antibodies to MuSK, but staining was unreliable in control muscles, so we cannot draw conclusions about its level or distribution in mutants. Interestingly, there appeared to be a clear correspondence between protein levels and AChRs intensity, such that the intensity of AChRs at a particular NMJ was correlated with that of the other component examined: high, mid, and low levels of AChRs were associated with high, mid, and low levels of the other synaptic proteins (data not shown).

### Agrin Loss Disrupts Nerve Terminal Organization

**Figure 3:**
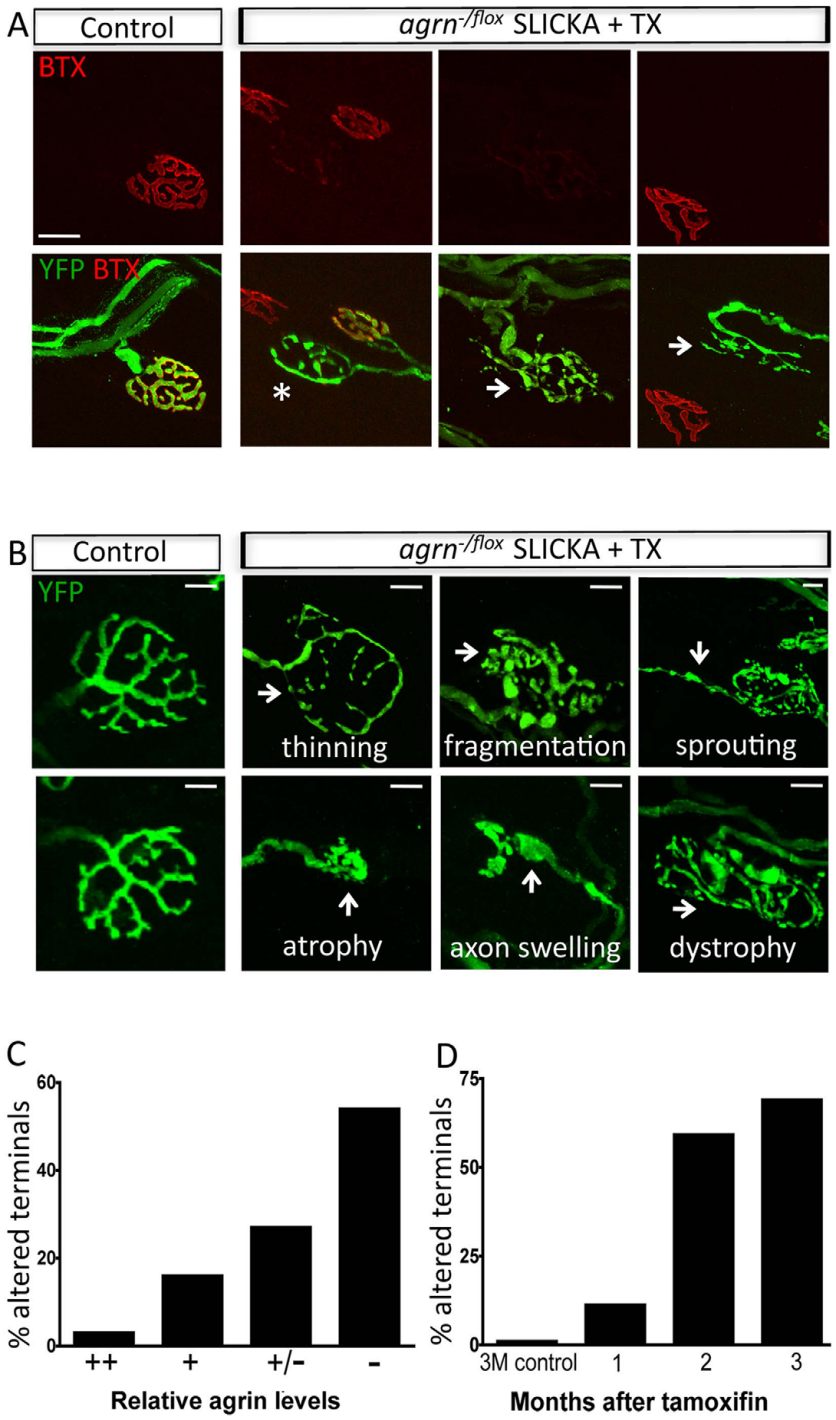
Agrin loss disrupts nerve terminal organization. A. Variation in nerve terminal morphology at AChR-poor NMJs in agrin mutants. Some terminals appear relatively normal (*) while others show marked dystrophies (arrows). Scale bar = 20 µm. B. Range of presynaptic defects in agrin mutant nerve terminals (arrows). YFP, green. C–D. The terminals that are affected show low levels of agrin relative to those that are unaffected (C), and the percentage of terminals that are altered increases over time (D). n = at least 4 samples per group.

To characterize presynaptic alterations at NMJs depleted of agrin, individual YFP+ nerve terminals from control and agrin mutant animals were imaged at a high magnification together with f-BTX at 3 months after tamoxifen injection (**Fig. 3A, B** **)**. Some YFP+ nerve terminals in mutants were apposed to postsynaptic sites containing normal levels of AChRs (**Fig. 3A**). The majority of these terminals were structurally normal, presumably because the *agrn* gene had not been disrupted in the parent neuron. Consistent with this idea, presynaptic alterations seen at 3 months after deletion occurred predominantly at NMJs from which agrin was depleted (**Fig. 3C**).

In contrast, terminals at NMJs with decreased AChR levels displayed presynaptic structural defects that ranged in severity from little alteration to severe disruption (**Fig. 3A**). Affected mutant terminals differed from those in control animals in several ways. In some cases, axon terminals displayed thinning and dystrophy, while in others the terminals showed fragmentation or swelling (**Fig. 3B**, arrows). Sprouting from a terminal occurred in rare cases. Together, these data suggest that agrin is required for maintaining AChR and axonal terminal patterning in adults.

In comparing pre- to postsynaptic alterations, we noted some cases in which relatively intact presynaptic structures were apposed to defective postsynaptic structures, but very few cases in which structurally abnormal nerve terminals apposed normal appearing postsynaptic structures. In addition, the majority of AChR aggregates in these muscles were unopposed by a YFP+ axon presumably because they were innervated by a YFP- axon. However, it was also possible that some AChR clusters had become denervated – that is, they had lost YFP+ innervation. To test this possibility, we stained all axons with antibodies to neurofilaments and found no endplates that lacked innervation (data not shown). Together, these results suggest that postsynaptic alterations precede presynaptic alterations.

To determine the sources of variability among affected nerve terminals, we performed three additional studies. First, we determined the fraction of YPF+ terminals with structural alterations at monthly intervals following tamoxifen administration. The percent of YFP+ terminals exhibiting one or more of the alterations described above increased from ∼12% at 1 month post deletion when agrin levels are fairly normal, to ∼75% at 3 months when agrin is largely depleted (**Fig. 3D**). As noted above, most of the YFP+ terminals that appeared normal at 3 months apposed agrin-positive postsynaptic sites, and therefore likely arose from motor neurons in which *agrn* had not been deleted.

Second, we examined several muscles that differ in position and fiber type composition. Changes in AChR clustering and nerve terminal architecture similar to those documented in Fig. 3 for triangularis sterni were observed in hindlimb, trunk and facial muscles (e.g. gastrocnemius, tibialis anterior, and levator aurius longus, and interscutularis; data not shown). Thus, most or all NMJs depend on agrin for their maintenance.

**Figure 4:**
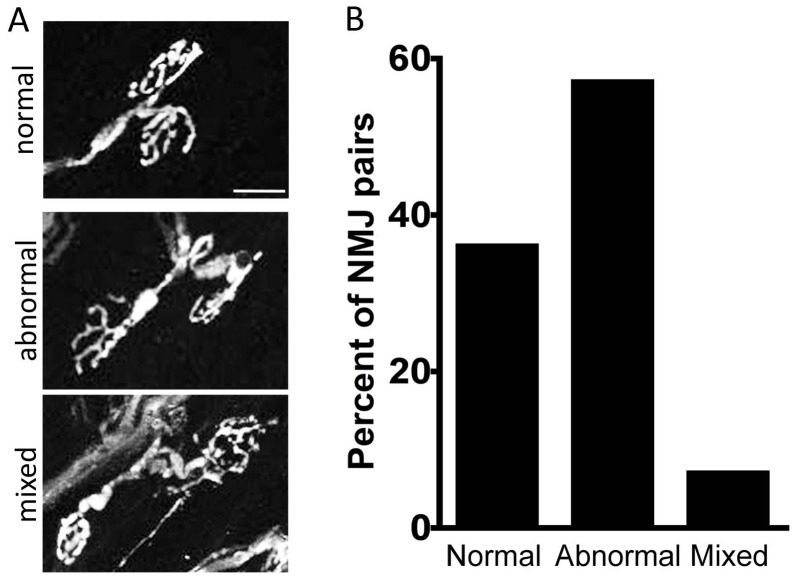
NMJs From the same motor neuron are similarly affected following conditional agrin deletion. A. Three pairs of nerve terminals from triangularis muscles of conditional agrin mutant 3 months after tamoxifen administration. In each case, both terminals arise from the same axon. Both are unaffected in one pair and both are affected in a second pair; in the third pair, one terminal is normal and the other is dystrophic. Scale bar = 20 µm. B. Quantification of nerve terminal pairs. n = 100 total pairs.

Third, we examined the variability in structural alterations among nerve terminals arising from a single motor axon. Because multiple YFP+ axons were present in most muscles, it was not feasible to reconstruct entire motor units, so we examined pairs of terminals in sparsely labeled regions that we knew arose from a single axon because we could trace them back to a common branch point. In nearly all cases the terminal structure of each member of a pair was similarly affected (**Fig. 4A**) and in <5% of pairs was one NMJ normal and the other clearly abnormal (**Fig. 4B**). These data support the idea that structural defects reflect the level of agrin within a motor unit and suggest that variation among NMJs may result from differences in the rate at which agrin is lost following deletion.

**Figure 5:**
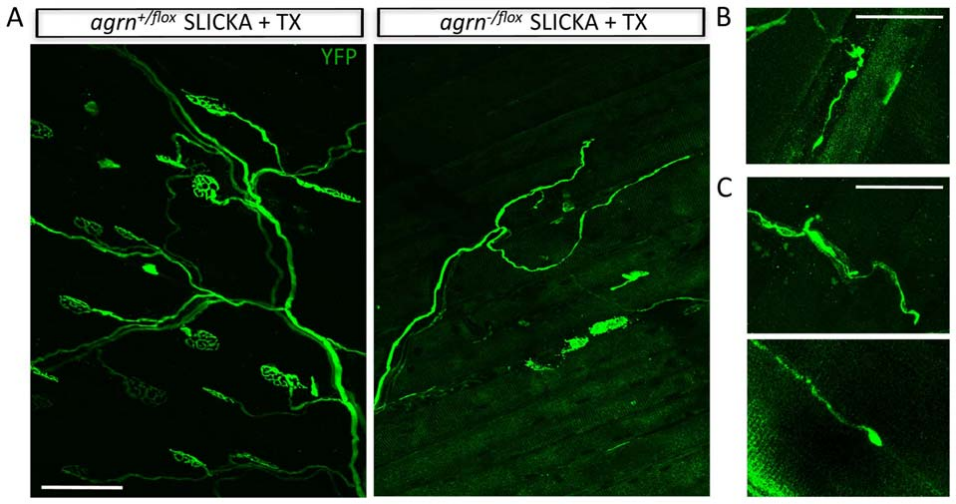
Nerve degeneration and death results from long-term agrin deletion. A. YFP labeled axons in the triangularis of control and agrin mutant mice 6 months after tamoxifen administration. Axons in the mutant show less branching than those in controls. Scale bar = 100 µm. B–C. Mutant nerve terminals show little to no presynaptic specialization (B) but rather often end in a single axon terminal bulb (C). Scale bar = 50 µm. YFP, green.

Finally, we extended our analysis to 5–6 months after tamoxifen injection to assess the fate of motor axons lacking agrin. By this time, pretzel shaped presynaptic nerve terminals had nearly all disappeared (**Fig. 5A**). The terminal structures that remained showed severely disrupted morphology with markedly simplified arbors; adjacent regions of muscle fibers were AChR-poor (**Fig. 5B**). In many cases, axons appeared to have withdrawn from synaptic sites, leaving a single fine-tipped or bulbous ending (**Fig. 5C**). Branching of YFP+ axons in mutants was much less complex than in control animals, leading to decreased size of the motor unit (**Fig. 5A** and data not shown). Moreover, the number of YFP+ axons per triangularis muscle was lower at 6 months after tamoxifen (1–3 per muscle) than at 1 or 3 months (4–10 per muscle). Thus, having lost their NMJs, the tamoxifen-deficient axons exhibit a “dying back” neuropathy typical of ALS and other axonal degeneration diseases [10].

### Presynaptic Alterations in NMJs of Laminin-α4 Mutants

**Figure 6:**
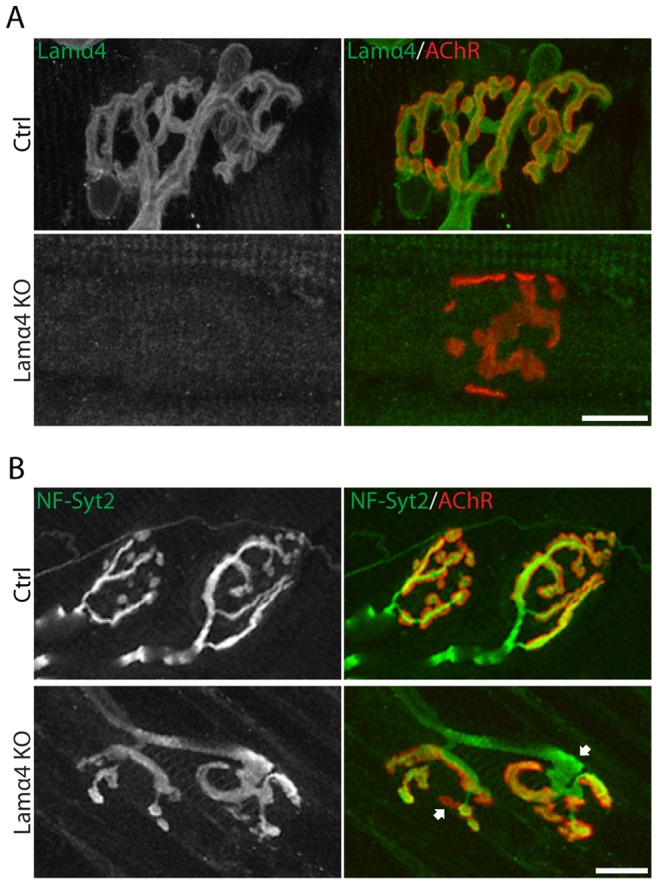
Abnormal NMJs in laminin-α4 knockout mice. A–B. Laminin-α4 is completely absent in the NMJs from 6 month old *lama4^−/−^* mice (A), and at this time point the NMJ has begun to atrophy (B). There are clear signs of axonal swellings (arrow) and denervation (star) in control 6 months old mice. Scale bar = 10 um.

To determine the role of muscle-derived organizers in synaptic maintenance we examined the role of laminin-α4 in adult NMJs. In previous studies, we showed that young adult *lama4^−/−^* mice are viable and fertile but have small NMJs in which active zones are misaligned with postsynaptic junctional folds [20]. Here, we asked whether additional alterations occurred at later ages. We stained muscles from control and *lama4^−/−^* mice with anti-neurofilaments to label axons, anti-synaptotagmin-2 to visualize nerve terminals, f-BTX to label AChRs, and anti-laminin-α4 to confirm deletion. At 40 days of age (sexual maturity), *lama4^−/−^* NMJs were smaller than those controls, but appeared largely normal by light microscopic criteria (data not shown; see ref [20]). At 6 months of age, however, nerve terminals were swollen and/or partially withdrawn from ∼50% of NMJs in *lama4^−/−^* mice (**Fig. 6**). In addition, whereas control NMJs are comprised of long branches, many mutant NMJs exhibited a fragmented appearance, with both nerve terminals and the AChR-rich postsynaptic apparatus broken into round or ovoid plaques.

**Figure 7:**
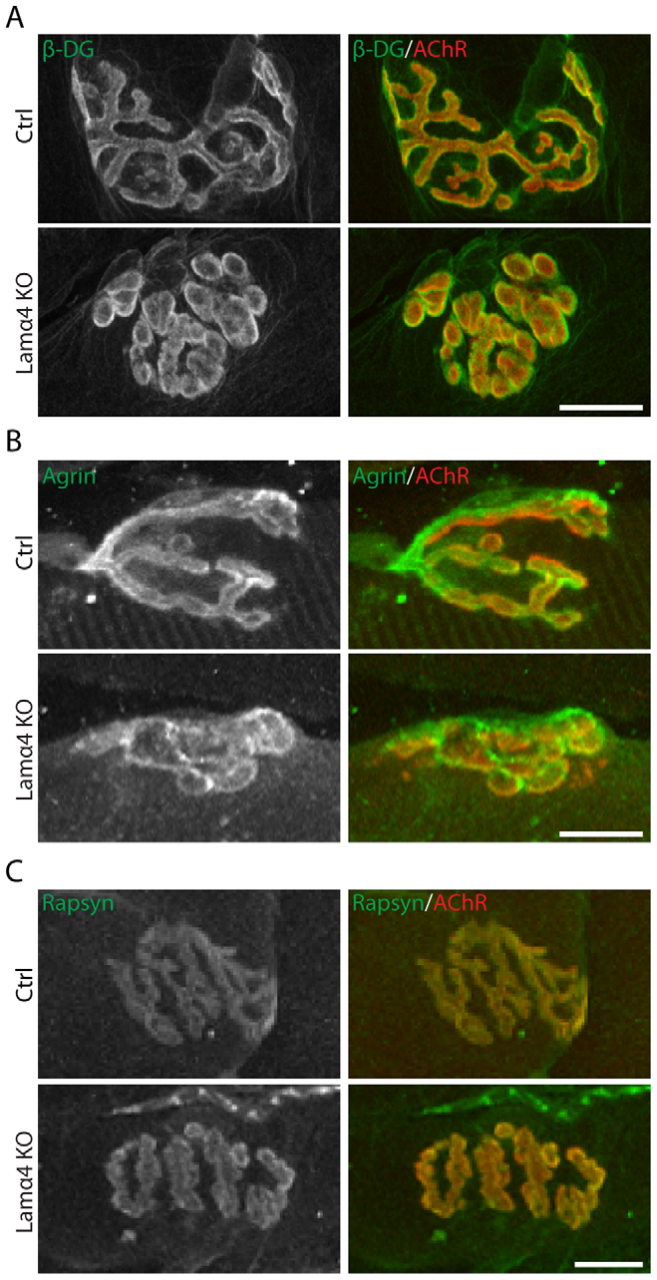
Deletion of laminin-α4 does not alter the levels or localization of other synaptic components. Distribution and levels of β-dystroglycan (A), agrin (B) and rapsyn (C) in 6 month old control and *lama4^−/−^* NMJs. All three proteins remain concentrated in NMJs lacking laminin-α4. Scale bar = 10 um.

To ask whether loss of laminin-α4 affected the molecular architecture as well as the geometry of the postsynaptic apparatus, we stained with a panel of antibodies to synaptic cleft and postsynaptic membrane components. We observed no marked difference between mutant and control NMJs in the levels of laminin-ß2, laminin-α5, agrin, ß-dystroglycan or rapsyn (**Fig. 7** and data not shown). The results are consistent with the idea that laminin-α4 exerts its effects directly on nerve terminals rather than regulating postsynaptic components directly and nerve terminals secondarily.

### Premature Aging of NMJs in Laminin-α4 Mutants

**Figure 8:**
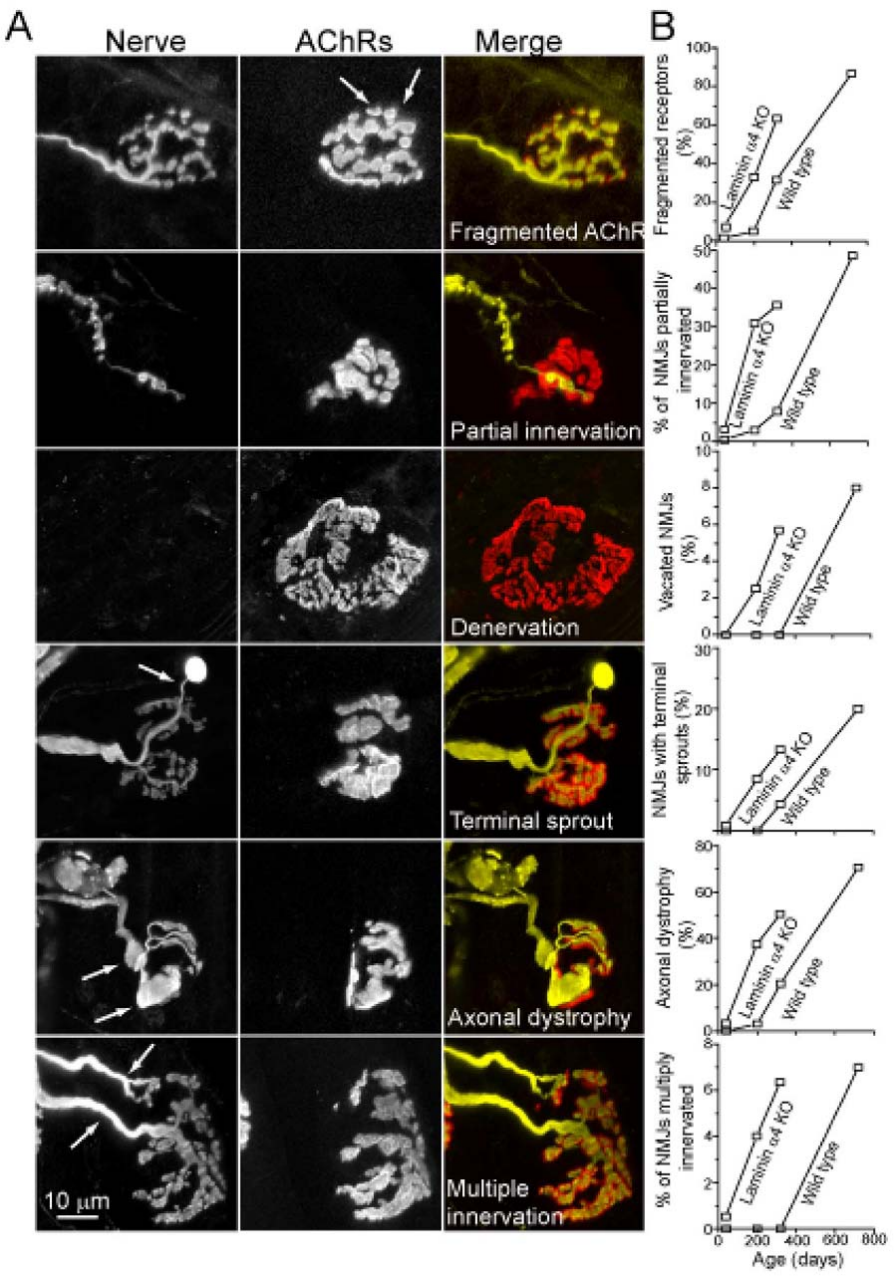
Absence of laminin-α4 causes NMJ to age prematurely. A. Axon terminals (left) and AChRs (middle panels) from diaphragm muscles of 6 month old *lama4^−/−^* mice. The age-associated abnormalities observed in mutants at 6 months of age were similar to those seen in two-year-old wild type mice. B. Prevalence of axonal and terminal changes in *lama4^−/−^* and wild type animals. At least 60 NMJs were analyzed for each genotype at each age. Scale Bar = 10 um.

The structural alterations observed in motor nerve terminals of 6 month-old *lama4* mutants resembled those observed in NMJs of 1.5–2 year old wild-type mice [8]. To quantify these alterations, we crossed *lama4* mutants to Thy1-YFP16 mice, which express YFP in all motoneurons [37]. We then determined the fraction of NMJs in diaphragm muscle that exhibited each of 6 features characteristic of aging NMJs: fragmentation of branches into small islands, partial or complete denervation of the postsynaptic membrane, terminal sprouting, axonal dystrophy and multiple innervation of individual synaptic sites (**Fig. 8A**). Results from control diaphragms were similar to those documented recently for hindlimb muscles [8]: the incidence of alterations was extremely low at 1 and 6 months of age, and then increased markedly between 1 and 2 years of age. In striking contrast, alterations were abundant in *lama4^−/−^* muscle at 6 months of age and increased further by 10 months of age (**Fig. 8B**). By these criteria, NMJs accumulated age-related alterations 6–10 months earlier in absence of laminin-α4 than in its presence.

### Alterations in the Synaptic Cleft of Aged NMJs

**Figure 9:**
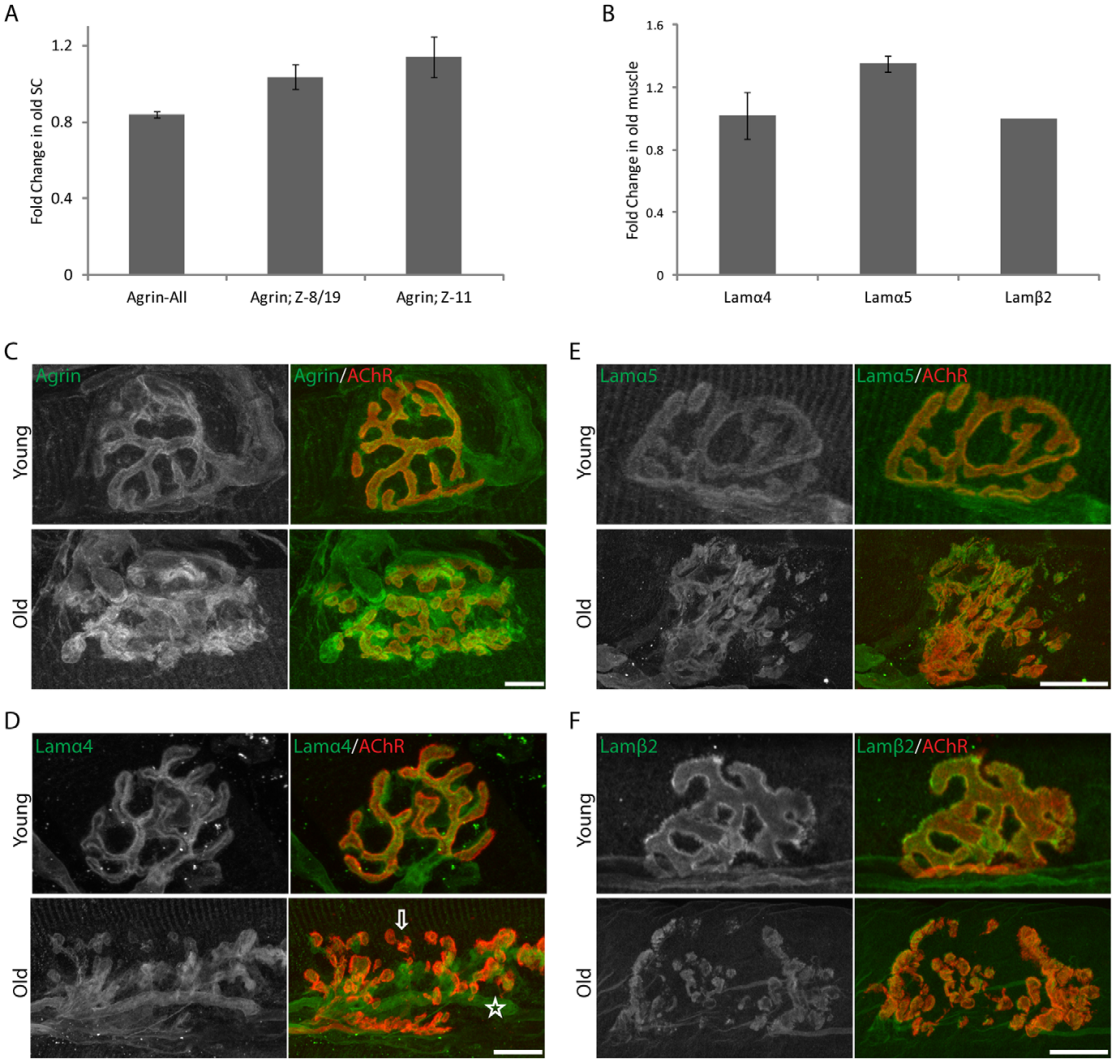
Molecular changes associated with aging neuromuscular junctions. A. Levels of RNA encoding different agrin isoforms were compared in the spinal cords of young adult and 2 year old mice by quantitative PCR. B. Levels of laminin-α4, -α5 and- β2 RNA were compared in young adult and 2 year old tibialis anterior muscles using quantitative PCR. C–F. Immunostaining with antibodies to agrin and three laminin subunits in young adult and old NMJs. Levels of these proteins did not change significantly with age, but their distribution was altered (see D bottom panel; star = extrasynaptic; arrow = diffuse or lack of expression). Bars represent the SEM. Scale Bar = 10 um.

Since deletion of agrin and laminin-α4 cause premature disassembly of the NMJ, we asked whether alterations in these molecules occur in aging NMJs. To test for transcriptional changes, we carried out quantitative PCR on samples from old and young mice using primers specific to different agrin isoforms and laminin-α4, -α5 and -β2 chains. We assayed agrin and laminin RNA levels in spinal cord and muscle, respectively, because the bioactive z-agrin in the synaptic cleft is derived exclusively from motoneurons whereas the synaptic laminins are synthesized in muscle fibers [38], [39]. The abundance of agrin mRNA (assayed with primers that amplify all isoforms) and z-agrin (assayed with exon-specific primers) did not differ detectably between spinal cords from young and old mice (**Fig. 9A**). Likewise, abundance of mRNAs encoding the three laminin chains changed little if at all in old muscles (**Fig. 9B**).

We next asked whether aging affected levels or distribution of these molecules at the NMJ. Young adult and aged whole triangularis muscles were stained with antibodies against agrin and the three synapse localized laminin chains. We detected no difference in levels of agrin or laminins between young adult and old muscles, but their distribution was markedly altered. Whereas all four proteins were precisely apposed to regions of high AChR density in young adults, some synaptic regions were depleted of these proteins in old mice, and immunoreactivity extended into perisynaptic areas (**Fig. 9C–F**).

## Discussion

A key feature of the adult NMJ is that it is remarkably stable under ordinary circumstances [1]–[3] yet capable of remodeling in response to altered circumstances such as increased load or denervation of nearby fibers [40]–[43]. This combination of stability and malleability implies that synaptic maintenance is controlled actively, yet little is known about the underlying molecular regulatory mechanisms. In this study, we used genetic methods to show that two synaptic organizing molecules known to regulate development of the NMJ, agrin and laminin-α4, are also required for synaptic maintenance in adults. We documented progressive presynaptic alterations in mice lacking laminin-α4 (*lama4* ^−/−^) and synaptic disassembly following deletion of agrin in adults (*agrn^flox/−^*;SLICKA). Alterations observed in the two mutants are related but distinct, and both resemble some of those seen during normal aging and in diseases of the motor unit [9]–[13]. We conclude that multiple synaptic organizers cooperate not only to form the NMJ during embryonic and early postembryonic life but also to maintain it in adults. Our results also raise the possibility that alterations in these or other organizing molecules are involved in age-dependent disassembly of the NMJ.

### Agrin is Required for Pre- and Postsynaptic Maintenance

By using a conditional agrin mutant (*agrn^flox^*), we could allow synapses to develop normally and then assess the effect of agrin loss in adulthood. A role for agrin in synaptic maintenance was not unexpected given its critical role in development [18], [19]. However, in the absence of innervation, and thus z-agrin, AChR aggregates form and other components of the postsynaptic membrane and synaptic cleft accumulate both in vitro and in vivo [26], [27], [44]. In contrast, the agrin co-receptor, MuSK is required for AChR aggregation under both normal and aneural conditions [22], [26], [27]. These and other results have led to a model in which basal levels of MuSK signaling initiate synapse formation, with agrin increasing MuSK activity to stabilize postsynaptic structures and induce formation of additional ones [15], [16]. Consistent with this idea, modest increases in MuSK levels in muscle, achieved transgenically, rescue the lethality of agrin mutants [45]. In view of these results, it was an open question whether agrin would be required to maintain appropriate AChR clustering in mature nerve terminals where other synaptic organizers remain.

Agrin depletion induced dramatic alterations at the NMJ: (1) AChR aggregates were lost from the postsynaptic membrane; (2) other components of the postsynaptic membrane (β-dystroglycan), subsynaptic cytoskeleton (rapsyn), and synaptic cleft (laminins-α4 and -α5) were depleted; and (3) nerve terminals remodeled, became dystrophic and eventually withdrew from the end-plate. Why does loss of agrin lead to so many structural alterations at the NMJ? The best-characterized role of agrin is to induce and stabilize AChR clusters. Other alterations could either reflect direct roles for agrin in many aspects of synaptic maintenance, or secondary effects of AChR loss. In favor of the latter possibility is the observation that postsynaptic defects appear to precede presynaptic ones: presynaptic alterations are found only at defective postsynaptic structures whereas postsynaptic alterations often occur at synapses with structurally normal nerve terminals. An attractive model is that dispersion of AChRs leads to rapid loss of other components, including presynaptic organizers such as ß2 laminins and LRP4 [46], which in turn leads more slowly to presynaptic changes. It is also possible, however, that agrin has multiple direct effects. In favor of this alternative, agrin treatment can cluster some synaptic proteins when applied to muscle cells that lack AChRs [47] and it can act directly on axons to affect growth, differentiation and adhesion [48]–[50]. Indeed, agrin-MuSK signaling is thought to encode an axon growth ‘stop signal’ as mutating either protein leads to exuberant axon sprouting [18], [19], [51]. It was perhaps surprising, then, that we only rarely detected axon sprouting in adult conditional agrin mutants. This may reflect either inherent differences in adult axon growth capacity or indicate that some functions of agrin are restricted to specific time windows.

The slow pace of change following *agrn* deletion presumably reflects the slow loss of z-agrin from the synaptic cleft. Although we have no data on the rate at which *agrn* mRNA and protein levels decline within motoneurons following gene inactivation, our results are consistent with the idea that agrin turns over slowly, as has been shown for other basal lamina components in many tissues, and for recombinant agrin injected into muscles [52]. Slow turnover could be an important factor in explaining the limited stability of postsynaptic sites following denervation. Although postsynaptic structures are maintained for weeks following denervation, AChR density subsequently declines and the postsynaptic apparatus disassembles in 2–3 months [53], a time consistent with the disappearance of agrin following *agrn* deletion.

### Laminin-α4 is Required for Maintenance of Nerve Terminals

To assess roles of synaptic laminins in maintenance of the NMJ, we examined mice lacking laminin-α4. Five laminin subunits are present in the basal lamina of the synaptic cleft -α2, α4, α5 and β2 and γ1- of which α4, α5 and β2 are present at several-fold higher levels synaptically than extrasynaptically. The rationale for analyzing α4 was as follows: lack of either broadly distributed subunit, α2 or γ1, has devastating effects on muscle integrity. Lack of β2 severely perturbs presynaptic development, but the null mutant is lethal [17] and no conditional mutant has been reported. Lack of α5 delays maturation of the NMJ but does not affect its adult form [34]. In contrast, laminin-α4 is required for precise apposition of pre- and postsynaptic structures at the NMJs in young adults, but does not detectably compromise the topology or molecular architecture of the synapse, and lama4^−/−^ mice are viable and fertile. We were therefore able to ask whether laminin-α4 is required for long-term maintenance of the NMJ as well as its earlier maturation.

In contrast to the complete synaptic disassembly that occurred following loss of neural agrin in adults, effects of deleting laminin-α4 were largely confined to presynaptic structures. Moreover, loss of laminin-α4 had no obvious effect on the molecular architecture of the synaptic cleft or postsynaptic membrane, either in young adults [20] or in 6–12-month old mice. Together, these data suggest that laminin-α4 acts directly on nerve terminals rather than indirectly via postsynaptic disruption or coordinate changes in the synaptic cleft. Laminin-α4 is concentrated in punctae within the synaptic cleft that directly appose active zones, and in young adult lama4^−/−^ mice, the normal precise apposition of active zones to junction folds is lost [20]. Thus laminin 421 may bind to receptors associated with active zones –for example, integrin α3 [54]. We speculate that loss of this link leads to decreased stability and eventual disassembly of the nerve terminal.

### Maintenance and Aging of the NMJ

NMJs react differently to the loss of agrin and laminin-α4. After agrin is deleted, specializations of the postsynaptic membrane and synaptic cleft are lost; presynaptic alterations occur later, perhaps, as discussed above, as an indirect consequence of the initial disruptions. In the absence of laminin-α4, in contrast, nerve terminals show gradual structural changes, but the synaptic cleft and postsynaptic membrane are spared. Thus, these two synaptic cleft proteins play distinct roles in synaptic maintenance. We draw two main conclusions from these data. First, synaptic organizing molecules, which are critical for embryonic and early postnatal formation of the NMJ, are also required for its long-term maintenance. Second, maintenance of the adult NMJ relies on combined effects of several distinct organizers.

Perhaps our most striking observation is that abnormalities observed in agrin or laminin-α4 mutants resemble those in aged wild-type mice. At present, we have no direct evidence on whether agrin or laminin are involved in the pathogenic mechanisms that underlie synaptic aging. However, the similarity between the mutant phenotypes and normal aging invites the speculation that alterations in these molecules could contribute to age-related synaptic disassembly. Three types of alterations could occur. First, levels of these components could decline with age. We see no dramatic decline, but our methods would not have allowed us to detect a modest decline. Second, the distribution of the proteins could be altered with age, decreasing their ability to mediate highly localized specializations of appositions. In fact, we have observed such alterations in distribution (Figure 9). Third, the molecular structure of the proteins could change with age. Consistent with this, both neurotrypsin and matrix metalloproteinase-3 (MMP-3) cleave agrin, and increased cleavage (or increased levels of cleavage fragments) has been proposed to promote age-related muscle decline, called sarcopenia [55]–[57]. Likewise, cleavage of laminin alpha chains occurs in vivo, and alters the bioactivities of the heterotrimers [58], [59]. Such cleavage could inactive agrin or laminins at the NMJ without affecting the epitopes that our antibodies detect and lead to protein redistribution yet not alter the total levels detectable by staining. A final possibility is that small changes in many different organizers, undetectable by our methods, could lead to age-related synaptic decline.

## Materials and Methods

### Mice

Generation and characterization of agrin null (*agrn^−/−^*) [18] agrin conditional (*agrn^flox/flox^*) [35], SlickA [36], and laminin α4 null (*lama4^−/−^*) mice [20] have been described previously. To conditionally delete agrin in a YFP marked subset of motor neurons, *agrn^+/−^* mice were mated to SLICKA animals to generate heterozygtes and the *agrn^+/−^;SlickA^+^* offspring were crossed to *agrn^flox/flox^* to generate transheterozygots carrying CreER (*agrn^−/flox^;* *SlickA*) as well as heterozygote controls (*agrn^+/+^*;*SlickA*). Tamoxifen dissolved in corn oil was administered over a two-week period by oral gavage once a day for 4 consecutive days during each week (∼3 mg/animal/dose).

For comparative studies of young and old mice, young adult (3 to 4 months) and aged (24 to 28 months) C57BL/6 mice were obtained from the National Institute on Aging or The Jackson Laboratory (Bar Harbor, Maine) or bred in our vivarium. All experiments conformed to NIH guidelines and were carried out in accordance with protocols approved by the Harvard University Standing Committee on the Use of Animals in Research and Teaching.

### qRT-PCR

For agrin and laminin transcripts analysis, total RNA was prepared from the spinal cords or skeletal muscles of young (3 to 4 months) and old (24 to 28 months) C57BL/6J mice using the RNEasy kit as per the manufacturer’s instructions (Qiagen, Valencia, VA). cDNA was generated using the QuantiTect Reverse Transcription system (Qiagen) and transcript levels were determined on a Stratagene MX3000p machine using SYBR green. Primers for the various z agrin splicing isoforms (total agrin, 8/19, 11) have been described and validated previously [60]. The following primers were used to detect laminin chains: β2 = GCTTGTAGAGGCCACAGAGG and GACCACTGAGTGCATGGTTG; α4 = TGTTTGTTGGAGGTGTTCCA and ATGCTCACAGCACCACTGAC; α5 = TACAACCTGTGACCCGACAA and GGATGACAAGAGCCAGCTTC.

### Immunohistochemistry

Mice were anesthetized with sodium pentobarbital and then transcardially perfused with phosphate buffered saline (PBS) followed by 4% paraformaldehyde (PFA) in PBS. Muscles were postfixed in PFA for 10 min and used for whole mount analysis.

For agrin staining, muscles were first exposed to ice cold methanol (100%) for 10 s and then washed in PBS. Tissue was then incubated successively with blocking solution (5% normal goat serum, 3% BSA and 0.5% Triton-X-100 in PBS) overnight at 4°C, primary antibodies diluted in blocking solution for 12–14 hours at 4°C, and fluorophore-labeled secondary antibodies or fBTX (Molecular Probes, Eugene, OR) for 2 to 3 hours at room temperature.

The primary antibodies used in this study were directed against the following molecules: agrin (1∶500, gift from Z. Hall); laminin-α4 and laminin-ß2 (1∶1000, gifts from T. Sasaki, Shriners Research Center, Portland OR); laminin-α5 (1∶1000, [61]); rapsyn (1∶250, Thermo), β-dystroglycan (1∶250, Developmental Studies Hybridoma Bank), GFP (1∶1000, Abcam); neurofilament (SMI312, 1∶1000, Covance); and syntaptotagmin 2 (Syt2, 1∶250, Developmental Studies Hybridoma Bank). Fluorescently labeled BTX (Alexa 488, 594, or 647; Invitrogen) was used to mark AChRs.

### Histological Analysis

Maximum intensity projections of confocal stacks were generated using Image J or Metamorph 6.03 software (Molecular Devices, Sunnyvale, CA). For structural analysis in *lama4^−/−^* mice, sprouting was defined as an axonal profile that extended beyond ≥2 µms of the endplate; partial or complete denervation was assayed by the apposition of the axon terminals and their AChRs; multiple innervation was identified by counting the number of axons impinging onto each endplate. For agrin conditional animals, altered NMJs were quantified as those that displayed any one of the several dystrophies outlined above, including axon swelling, nerve sprouting, atrophy, fragmentation, and thinning. For axon terminal pair analyses, whole triangularis muscles from conditional agrin mice were imaged and reconstructed. Images were scanned to identify those in which two terminals arose from the same axon and their level of dystrophy was then evaluated relative to control animals.

## References

[1] Lichtman JW, Magrassi L, Purves D (1987) Visualization of neuromuscular junctions over periods of several months in living mice. J Neurosci 7: 1215–1222. 3572477 10.1523/JNEUROSCI.07-04-01215.1987 PMC6569015

[2] Wigston DJ (1990) Repeated in vivo visualization of neuromuscular junctions in adult mouse lateral gastrocnemius. J Neurosci 10: 1753–1761. 2355249 10.1523/JNEUROSCI.10-06-01753.1990 PMC6570303

[3] Balice-Gordon RJ, Lichtman JW (1990) In vivo visualization of the growth of pre- and postsynaptic elements of neuromuscular junctions in the mouse. J Neurosci 10: 894–908. 2156964 10.1523/JNEUROSCI.10-03-00894.1990 PMC6570117

[4] Balice-Gordon RJ (1997) Age-related changes in neuromuscular innervation. Muscle Nerve Suppl 5: S83–87. 10.1002/(sici)1097-4598(1997)5+<83::aid-mus20>3.0.co;2-z 9331392

[5] Cardasis CA, LaFontaine DM (1987) Aging rat neuromuscular junctions: a morphometric study of cholinesterase-stained whole mounts and ultrastructure. Muscle Nerve 10: 200–213. 2436045 10.1002/mus.880100303

[6] Fahim MA, Robbins N (1982) Ultrastructural studies of young and old mouse neuromuscular junctions. J Neurocytol 11: 641–656. 7131048 10.1007/BF01262429

[7] Fahim MA, Holley JA, Robbins N (1983) Scanning and light microscopic study of age changes at a neuromuscular junction in the mouse. J Neurocytol 12: 13–25. 6842270 10.1007/BF01148085

[8] Valdez G, Tapia JC, Kang H, Clemenson GD Jr, Gage FH, et al (2010) Attenuation of age-related changes in mouse neuromuscular synapses by caloric restriction and exercise. Proc Natl Acad Sci U S A 107: 14863–14868. 20679195 10.1073/pnas.1002220107 PMC2930485

[9] Valdez G, Tapia JC, Lichtman JW, Fox MA, Sanes JR (2012) Shared resistance to aging and ALS in neuromuscular junctions of specific muscles. PLoS One 7: e34640 doi: 10.1371/journal.pone.0034640. 22485182 10.1371/journal.pone.0034640 PMC3317643

[10] Fischer LR, Culver DG, Tennant P, Davis AA, Wang M, et al (2004) Amyotrophic lateral sclerosis is a distal axonopathy: evidence in mice and man. Exp Neurol 185: 232–240. 14736504 10.1016/j.expneurol.2003.10.004

[11] Gurney ME, Pu H, Chiu AY, Dal Canto MC, Polchow CY, et al (1994) Motor neuron degeneration in mice that express a human Cu,Zn superoxide dismutase mutation. Science 264: 1772–1775. 8209258 10.1126/science.8209258

[12] Gomez AM, Van Den Broeck J, Vrolix K, Janssen SP, Lemmens MA, et al (2010) Antibody effector mechanisms in myasthenia gravis-pathogenesis at the neuromuscular junction. Autoimmunity 43: 353–370. 20380584 10.3109/08916930903555943

[13] Schaefer AM, Sanes JR, Lichtman JW (2005) A compensatory subpopulation of motor neurons in a mouse model of amyotrophic lateral sclerosis. J Comp Neurol 490: 209–219. 16082680 10.1002/cne.20620

[14] Sanes JR, Lichtman JW (2001) Induction, assembly, maturation and maintenance of a postsynaptic apparatus. Nat Rev Neurosci 2: 791–805. 11715056 10.1038/35097557

[15] Kummer TT, Misgeld T, Sanes JR (2006) Assembly of the postsynaptic membrane at the neuromuscular junction: paradigm lost. Curr Opin Neurobiol 16: 74–82. 16386415 10.1016/j.conb.2005.12.003

[16] Wu H, Xiong WC, Mei L (2010) To build a synapse: signaling pathways in neuromuscular junction assembly. Development 137: 1017–1033. 20215342 10.1242/dev.038711 PMC2835321

[17] Noakes PG, Gautam M, Mudd J, Sanes JR, Merlie JP (1995) Aberrant differentiation of neuromuscular junctions in mice lacking s-laminin/laminin beta 2. Nature 374: 258–262. 7885444 10.1038/374258a0

[18] Gautam M, Noakes PG, Moscoso L, Rupp F, Scheller RH, et al (1996) Defective neuromuscular synaptogenesis in agrin-deficient mutant mice. Cell 85: 525–535. 8653788 10.1016/s0092-8674(00)81253-2

[19] Burgess RW, Nguyen QT, Son YJ, Lichtman JW, Sanes JR (1999) Alternatively spliced isoforms of nerve- and muscle-derived agrin: their roles at the neuromuscular junction. Neuron 23: 33–44. 10402191 10.1016/s0896-6273(00)80751-5

[20] Patton BL, Cunningham JM, Thyboll J, Kortesmaa J, Westerblad H, et al (2001) Properly formed but improperly localized synaptic specializations in the absence of laminin alpha4. Nat Neurosci 4: 597–604. 11369940 10.1038/88414

[21] Fox MA, Sanes JR, Borza DB, Eswarakumar VP, Fassler R, et al (2007) Distinct target-derived signals organize formation, maturation, and maintenance of motor nerve terminals. Cell 129: 179–193. 17418794 10.1016/j.cell.2007.02.035

[22] DeChiara TM, Bowen DC, Valenzuela DM, Simmons MV, Poueymirou WT, et al (1996) The receptor tyrosine kinase MuSK is required for neuromuscular junction formation in vivo. Cell 85: 501–512. 8653786 10.1016/s0092-8674(00)81251-9

[23] Glass DJ, Bowen DC, Stitt TN, Radziejewski C, Bruno J, et al (1996) Agrin acts via a MuSK receptor complex. Cell 85: 513–523. 8653787 10.1016/s0092-8674(00)81252-0

[24] Kim N, Stiegler AL, Cameron TO, Hallock PT, Gomez AM, et al (2008) Lrp4 is a receptor for Agrin and forms a complex with MuSK. Cell 135: 334–342. 18848351 10.1016/j.cell.2008.10.002 PMC2933840

[25] Zhang B, Luo S, Wang Q, Suzuki T, Xiong WC, et al (2008) LRP4 serves as a coreceptor of agrin. Neuron 60: 285–297. 18957220 10.1016/j.neuron.2008.10.006 PMC2743173

[26] Yang X, Arber S, William C, Li L, Tanabe Y, et al (2001) Patterning of muscle acetylcholine receptor gene expression in the absence of motor innervation. Neuron 30: 399–410. 11395002 10.1016/s0896-6273(01)00287-2

[27] Lin W, Burgess RW, Dominguez B, Pfaff SL, Sanes JR, et al (2001) Distinct roles of nerve and muscle in postsynaptic differentiation of the neuromuscular synapse. Nature 410: 1057–1064. 11323662 10.1038/35074025

[28] Lin W, Dominguez B, Yang J, Aryal P, Brandon EP, et al (2005) Neurotransmitter acetylcholine negatively regulates neuromuscular synapse formation by a Cdk5-dependent mechanism. Neuron 46: 569–579. 15944126 10.1016/j.neuron.2005.04.002

[29] Misgeld T, Kummer TT, Lichtman JW, Sanes JR (2005) Agrin promotes synaptic differentiation by counteracting an inhibitory effect of neurotransmitter. Proc Natl Acad Sci U S A 102: 11088–11093. 16043708 10.1073/pnas.0504806102 PMC1182450

[30] Flanagan-Steet H, Fox MA, Meyer D, Sanes JR (2005) Neuromuscular synapses can form in vivo by incorporation of initially aneural postsynaptic specializations. Development 132: 4471–4481. 16162647 10.1242/dev.02044

[31] Miner JH, Yurchenco PD (2004) Laminin functions in tissue morphogenesis. Annu Rev Cell Dev Biol 20: 255–284. 15473841 10.1146/annurev.cellbio.20.010403.094555

[32] Patton BL, Miner JH, Chiu AY, Sanes JR (1997) Distribution and function of laminins in the neuromuscular system of developing, adult, and mutant mice. J Cell Biol 139: 1507–1521. 9396756 10.1083/jcb.139.6.1507 PMC2132624

[33] Patton BL, Chiu AY, Sanes JR (1998) Synaptic laminin prevents glial entry into the synaptic cleft. Nature 393: 698–701. 9641682 10.1038/31502

[34] Nishimune H, Valdez G, Jarad G, Moulson CL, Muller U, et al (2008) Laminins promote postsynaptic maturation by an autocrine mechanism at the neuromuscular junction. J Cell Biol 182: 1201–1215. 18794334 10.1083/jcb.200805095 PMC2542479

[35] Harvey SJ, Jarad G, Cunningham J, Rops AL, van der Vlag J, et al (2007) Disruption of glomerular basement membrane charge through podocyte-specific mutation of agrin does not alter glomerular permselectivity. Am J Pathol 171: 139–152. 17591961 10.2353/ajpath.2007.061116 PMC1941581

[36] Young P, Qiu L, Wang D, Zhao S, Gross J, et al (2008) Single-neuron labeling with inducible Cre-mediated knockout in transgenic mice. Nat Neurosci 11: 721–728. 18454144 10.1038/nn.2118 PMC3062628

[37] Feng G, Mellor RH, Bernstein M, Keller-Peck C, Nguyen QT, et al (2000) Imaging neuronal subsets in transgenic mice expressing multiple spectral variants of GFP. Neuron 28: 41–51. 11086982 10.1016/s0896-6273(00)00084-2

[38] Hoch W, Ferns M, Campanelli JT, Hall ZW, Scheller RH (1993) Developmental regulation of highly active alternatively spliced forms of agrin. Neuron 11: 479–490. 8398141 10.1016/0896-6273(93)90152-h

[39] Sanes JR (2003) The basement membrane/basal lamina of skeletal muscle. J Biol Chem 278: 12601–12604. 12556454 10.1074/jbc.R200027200

[40] Balice-Gordon RJ, Breedlove SM, Bernstein S, Lichtman JW (1990) Neuromuscular junctions shrink and expand as muscle fiber size is manipulated: in vivo observations in the androgen-sensitive bulbocavernosus muscle of mice. J Neurosci 10: 2660–2671. 2388082 10.1523/JNEUROSCI.10-08-02660.1990 PMC6570280

[41] Brown MC, Holland RL, Hopkins WG (1981) Motor nerve sprouting. Annu Rev Neurosci 4: 17–42. 7013635 10.1146/annurev.ne.04.030181.000313

[42] Andonian MH, Fahim MA (1988) Endurance exercise alters the morphology of fast- and slow-twitch rat neuromuscular junctions. Int J Sports Med 9: 218–223. 3410628 10.1055/s-2007-1025009

[43] Deschenes MR, Tenny KA, Wilson MH (2006) Increased and decreased activity elicits specific morphological adaptations of the neuromuscular junction. Neuroscience 137: 1277–1283. 16359818 10.1016/j.neuroscience.2005.10.042

[44] Sanes JR, Feldman DH, Cheney JM, Lawrence JC Jr (1984) Brain extract induces synaptic characteristics in the basal lamina of cultured myotubes. J Neurosci 4: 464–473. 6366153 10.1523/JNEUROSCI.04-02-00464.1984 PMC6564900

[45] Kim N, Burden SJ (2008) MuSK controls where motor axons grow and form synapses. Nat Neurosci 11: 19–27. 18084289 10.1038/nn2026 PMC2923649

[46] Yumoto N, Kim N, Burden SJ (2012). Lrp4 Is a retrograde signal for presynaptic differentiation at neuromuscular synapses. Nature doi:10.1038/nature11348. 10.1038/nature11348 PMC3448831 22854782

[47] Marangi PA, Forsayeth JR, Mittaud P, Erb-Vogtli S, Blake DJ, et al (2001) Acetylcholine receptors are required for agrin-induced clustering of postsynaptic proteins. EMBO J 20: 7060–7073. 11742983 10.1093/emboj/20.24.7060 PMC125801

[48] Campagna JA, Ruegg MA, Bixby JL (1997) Evidence that agrin directly influences presynaptic differentiation at neuromuscular junctions in vitro. Eur J Neurosci 9: 2269–2283. 9464922 10.1111/j.1460-9568.1997.tb01645.x

[49] Burgess RW, Dickman DK, Nunez L, Glass DJ, Sanes JR (2002) Mapping sites responsible for interactions of agrin with neurons. J Neurochem 83: 271–284. 12423238 10.1046/j.1471-4159.2002.01102.x

[50] Wu H, Lu Y, Shen C, Patel N, Gan L, et al (2012) Distinct Roles of Muscle and Motoneuron LRP4 in Neuromuscular Junction Formation. Neuron 75: 94–107. 22794264 10.1016/j.neuron.2012.04.033 PMC3422364

[51] Hesser BA, Henschel O, Witzemann V (2006) Synapse disassembly and formation of new synapses in postnatal muscle upon conditional inactivation of MuSK. Mol Cell Neurosci 31: 470–480. 16337809 10.1016/j.mcn.2005.10.020

[52] Bezakova G, Helm JP, Francolini M, Lomo T (2001) Effects of purified recombinant neural and muscle agrin on skeletal muscle fibers in vivo. J Cell Biol 153: 1441–1452. 11425874 10.1083/jcb.153.7.1441 PMC2150725

[53] Miyamaru S, Kumai Y, Ito T, Yumoto E (2008) Effects of long-term denervation on the rat thyroarytenoid muscle. Laryngoscope 118: 1318–1323. 18425051 10.1097/MLG.0b013e31816f693f

[54] Cohen MW, Hoffstrom BG, DeSimone DW (2000) Active zones on motor nerve terminals contain alpha 3beta 1 integrin. J Neurosci 20: 4912–4921. 10864949 10.1523/JNEUROSCI.20-13-04912.2000 PMC6772282

[55] Bolliger MF, Zurlinden A, Luscher D, Butikofer L, Shakhova O, et al (2010) Specific proteolytic cleavage of agrin regulates maturation of the neuromuscular junction. J Cell Sci 123: 3944–3955. 20980386 10.1242/jcs.072090

[56] Butikofer L, Zurlinden A, Bolliger MF, Kunz B, Sonderegger P (2011) Destabilization of the neuromuscular junction by proteolytic cleavage of agrin results in precocious sarcopenia. FASEB J 25: 4378–4393. 21885656 10.1096/fj.11-191262

[57] Werle MJ, VanSaun M (2003) Activity dependent removal of agrin from synaptic basal lamina by matrix metalloproteinase 3. J Neurocytol 32: 905–913. 15034275 10.1023/B:NEUR.0000020631.69804.f5

[58] Bair EL, Chen ML, McDaniel K, Sekiguchi K, Cress AE, et al (2005) Membrane type 1 matrix metalloprotease cleaves laminin-10 and promotes prostate cancer cell migration. Neoplasia 7: 380–389. 15967115 10.1593/neo.04619 PMC1501144

[59] Baudoin C, Fantin L, Meneguzzi G (2005) Proteolytic processing of the laminin alpha3 G domain mediates assembly of hemidesmosomes but has no role on keratinocyte migration. J Invest Dermatol 125: 883–888. 16297184 10.1111/j.0022-202X.2005.23881.x

[60] Ruggiu M, Herbst R, Kim N, Jevsek M, Fak JJ, et al (2009) Rescuing Z+ agrin splicing in Nova null mice restores synapse formation and unmasks a physiologic defect in motor neuron firing. Proc Natl Acad Sci U S A 106: 3513–8. 19221030 10.1073/pnas.0813112106 PMC2642475

[61] Miner JH, Patton BL, Lentz SI, Gilbert DJ, Snider WD, et al (1997) The laminin alpha chains: expression, developmental transitions, and chromosomal locations of alpha1–5, identification of heterotrimeric laminins 8–11, and cloning of a novel alpha3 isoform. J Cell Biol 137: 685–701. 9151674 10.1083/jcb.137.3.685 PMC2139892

